# Supplementary material for: Fast and Sustained Axonal Growth by BDNF Released from Chitosan Microspheres
Source: Mar Drugs. 2023 Jan 27;21(2):91. doi: 10.3390/md21020091 (PMC9959400; doi:10.3390/md21020091)
Supplement: Supplementary file 1 [file marinedrugs-21-00091-s001.zip › marinedrugs-2153515-supplementary.pdf]

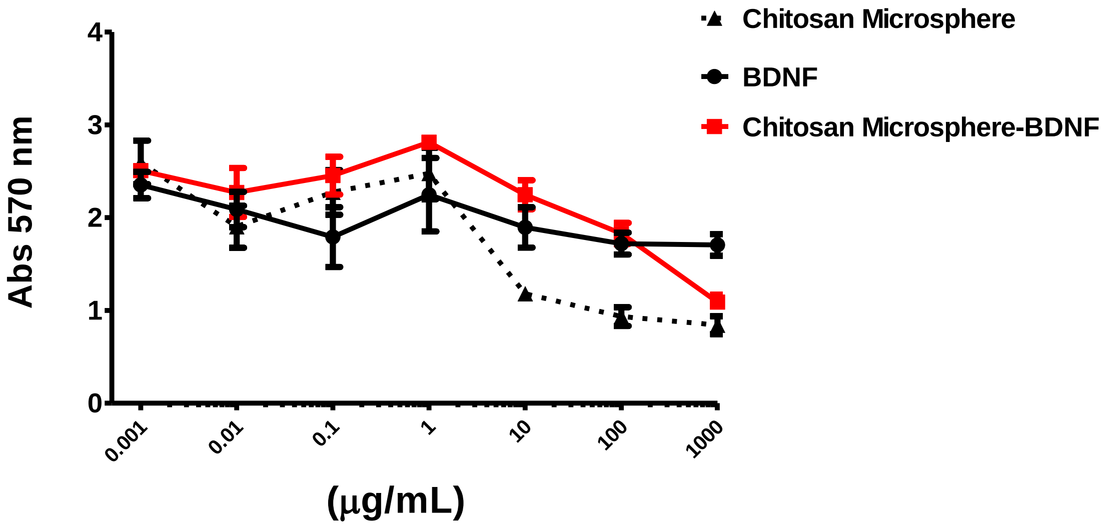

Figure S1. Cytotoxicity of BDNF-loaded chitosan microspheres in PC12 cells. The MTT viability assay was performed to follow the metabolism of PC12 cells incubated for 3 days with experimental variants as shown. There were not significant differences between chitosan microsphere-BDNF treatment and controls (ANOVA, $p = 0.34$).
